# Understanding tree growth responses after partial cuttings: A new approach

**DOI:** 10.1371/journal.pone.0172653

**Published:** 2017-02-21

**Authors:** Miguel Montoro Girona, Sergio Rossi, Jean-Martin Lussier, Denis Walsh, Hubert Morin

**Affiliations:** 1 Département des Sciences Fondamentales, Université du Québec à Chicoutimi, Chicoutimi, Québec, Canada; 2 Key Laboratory of Vegetation Restoration and Management of Degraded Ecosystems, Provincial Key Laboratory of Applied Botany, South China Botanical Garden, Chinese Academy of Sciences, Guangzhou, China; 3 Canadian Wood Fibre Centre, Canadian Forest Service, Natural Resources Canada, Quebec, Québec, Canada; Chinese Academy of Forestry, CHINA

## Abstract

Forest ecosystem management heads towards the use of partial cuttings. However, the wide variation in growth response of residual trees remains unexplained, preventing a suitable prediction of forest productivity. The aim of the study was to assess individual growth and identify the driving factors involved in the responses of residual trees. Six study blocks in even-aged black spruce [*Picea mariana* (Mill.) B.S.P.] stands of the eastern Canadian boreal forest were submitted to experimental shelterwood and seed-tree treatments. Individual-tree models were applied to 1039 trees to analyze their patterns of radial growth during the 10 years after partial cutting by using the nonlinear Schnute function on tree-ring series. The trees exhibited different growth patterns. A sigmoid growth was detected in 32% of trees, mainly in control plots of older stands. Forty-seven percent of trees located in the interior of residual strips showed an S-shape, which was influenced by stand mortality, harvested intensity and dominant height. Individuals showing an exponential pattern produced the greatest radial growth after cutting and were edge trees of younger stands with higher dominant height. A steady growth decline was observed in 4% of trees, represented by the individuals suppressed and insensitive to the treatment. The analyses demonstrated that individual nonlinear models are able to assess the variability in growth within the stand and the factors involved in the occurrence of the different growth patterns, thus improving understanding of the tree responses to partial cutting. This new approach can sustain forest management strategies by defining the best conditions to optimize the growth yield of residual trees.

## Introduction

Forest ecosystem management proposes partial cuttings as an alternative to achieve the sustainability of forestry in the boreal regions [1–3]. Partial cuttings integrate ecological and economic factors such as stand growth, tree quality, product yields [4, 5], and increase the habitat for wildlife by maintaining the overstory residual cover [3]. Consequently, the use of partial cuttings in silviculture is increasing in North America, and in particular in Eastern Canada [6]. However, investigations are needed to combine the best treatment for each species according to their ecological requirements, in order to maximize radial growth of the residual trees and enhance their economic value for lumber production. Forests are dynamic and complex systems, involving a number of ecological factors and processes interacting at multiples scales. It is thus necessary to develop tools that consider the spatio-temporal heterogeneity in growth, including nonlinear responses to the environment [7]. Tree growth models contribute to quantifying forest productivity, and are decision-support tools in sustainable forest management [8]. However, the traditional models have often simplified the growth response to environmental factors by assuming linear relationships between variables [9–11]. The growth response has a mostly sigmoid form, and more appropriate methods should be chosen to describe these complex biological mechanisms e.g. individual nonlinear approaches [12, 13]. Individual-tree models allow the growth process to be simulated under different experimental management regimes [14].

There is high diversity in the approaches and multiplicity in the forms of nonlinear growth functions [8], and some of them, like the Charman-Richard, Weibull and Schnute functions, have demonstrated good performance [15–19]. At the moment, the majority of studies on nonlinear growth modelling in the boreal forest have focused on the height and diameter relationship using inventory data to estimate the timber volume and growth yield [8, 16, 20, 21], climate and growth relationship [22], and stand structure in the context of natural succession [15]. Although, an increasing effort has been made to develop individual-tree diameter growth models, this has been limited in boreal forests, especially after partial cutting [20]. The majority of growth studies are based on the traditional forest inventories with diameter measurements, and nonlinear models based on tree-ring chronologies are not common. Tree-ring series provide more accurate estimations of radial growth than inventories data because they allow reconstruction at fine resolution [23]. Consequently, developing a new approach to study the growth response after partial cutting with dendroecological data and using nonlinear functions would be a major contribution in forest science.

Growth can vary greatly among trees, and stand and individual characteristics play a crucial role in this variation [24]. Tree-growth is related to stand development, and mediated by age-structure [25, 26], basal area [27, 28], and neighbour tree mortality [29]. In the case of individual variables, it has been demonstrated that inter-tree competition [24, 29, 30] and microclimate [31] affects growth, being influenced by the tree spatial position [29, 32], and its status, represented by the crown length [33] and tree diameter [20, 34]. As these variables are heterogeneous within a stand, understanding the variability in the growth response among trees clearly requires the application of individual-tree models.

Black spruce [*Picea mariana* (Mill.) B.S.P.] is one of the most important commercial trees in North-America because of its transcontinental distribution and wood properties such as high density, elasticity, resistance and fibre length [35–37]. Thanks to its high plasticity, black spruce grows in broad environmental conditions and latitudes [38], ranging from sea level to 1500 m [39] enduring extreme stress situations [40]. Black spruce is the main species in the eastern—Canadian boreal forest, representing approximately 75% of the total gross merchantable volume [36], and its wood is highly valued by the industry [41]. Despite the advances in the knowledge related to the growth responses of this species to partial cuttings, 50% of growth in residual trees remains unexplained [25, 26, 32, 42, 43]. The abovementioned studies suggest that the heterogeneity in growth response is due to soil conditions, root formation, or spatial variation, but with the current state of knowledge, these remain hypotheses. As a result, an important part of the variability in the individual growth response of trees after partial cuttings remains unaddressed. Deeper investigations are thus necessary to understand the factors involved in the heterogeneity of tree-growth due to the important implications for forest management to improve the forecast accuracy of growth models, maximize radial growth yield (e.g. stand selection), and adapt these treatments to boreal conditions in order to maintain the sustainability of North-American forestry.

In this study, we propose a new analytic tool to characterize and analyse individual radial growth by using the nonlinear Schnute function. The aim of our approach is to (i) develop individual models of growth response after partial cuttings; (ii) identify the driving factors influencing the frequency of the different tree growth patterns.

## Materials and methods

The "Ministère des Forêts, de la Faune et des Parcs (MFFP)" of Quebec provided the specific permissions necessary to develop our research, supervised the project and contributed to the funding by a "Fonds de recherche du Québec –Nature et technologies (FRQNT)" subvention. Authors confirm that the study did not involve endangered or protected species.

### Study area

**Fig 1:**
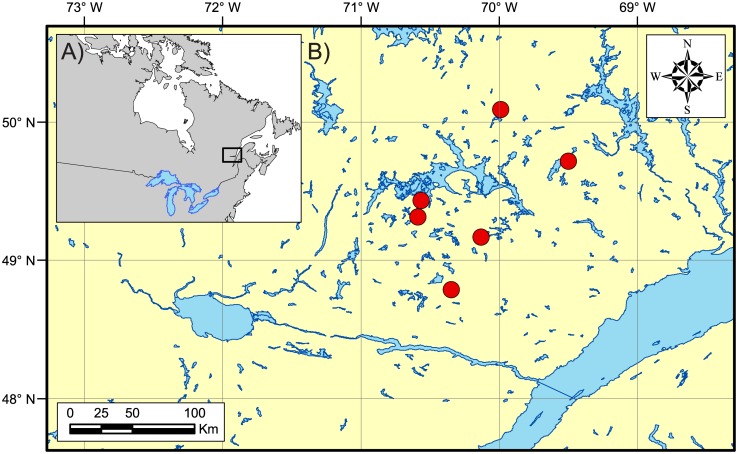
Geographic location of the study area in North America (A) and the experimental blocks (B).

The study was conducted in natural boreal forest stands of the Monts-Valin and North-Shore regions of Quebec, Canada (Fig 1A). These regions represent the main area of forest exploitation, and the stands were selected for their high productivity. The study area includes two bioclimatic zones: the balsam fir [*Abies balsamea* Mill.]–white birch (*Betula papyrifera* Marsh.) and the eastern spruce–feathermosses [44] (Fig 1B). The climate is subhumid subpolar, characterized by a short vegetation season of 140 days [45]. Annual temperature ranges between -2 and 1.5°C, with annual precipitation of 950–1350 mm [46]

### Experimental design

In 2003, the Canadian Forest Service performed a factorial experiment with completely randomized blocks in mature even-aged stands dominated by black spruce to assess the tree growth response after experimental partial cuttings [47]. Two structure types were selected: young and low-regenerated dense stands (80–100 years, 2600 trees/ha), and old and well-regenerated open stands (120–150 years, 1500 trees/ha). Six blocks were sampled, each one including five experimental units with a replicate of each silvicultural treatment and an untreated control (30 plots). The experimental units consisted of square permanent plots of 3 ha, and were relatively homogeneous and comparable within the same block in terms of composition and density. Tree spatial position in the residual strips was considered in two classes: edge or interior, the edge being an area of 1.25 m in width close to the skidding trails. The treatments and tree spatial positions represented the experimental factors arranged in a 4×2 factorial design with a control (S1 Appendix).

### Silvicultural treatments

**Fig 2:**
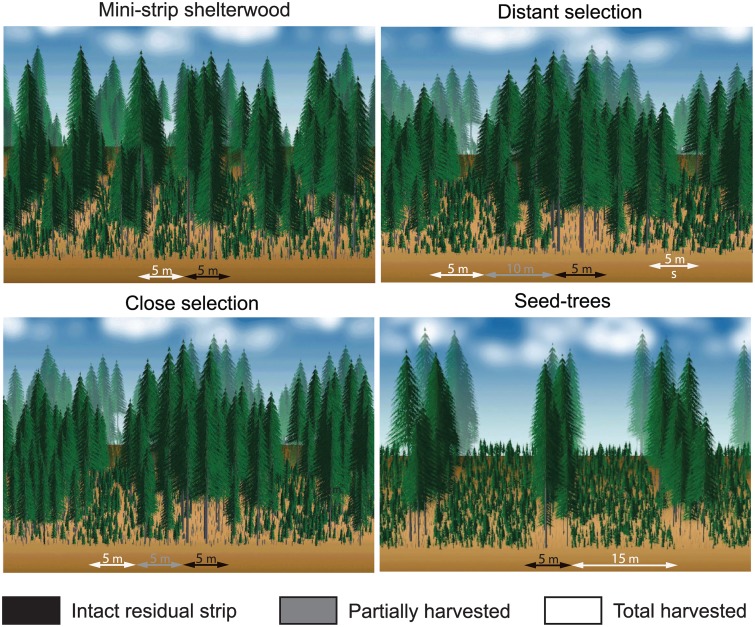
Characteristics and trail layout of the four treatments. White arrows represent total harvested surface or skidding trails, grey and black arrows indicate the surface of the partially harvested residual strip, and intact residual strip, respectively. The secondary trail is marked with the letter S.

Four cutting treatments were performed with single-grip harvesters and forwarders: mini-strip shelterwood (MS), distant selection (DS), close selection (CS) and seed-trees (ST). The first three treatments were variants of the uniform shelterwood system applied to promote regeneration in mature even-aged stands with a uniform opening of the canopy [48]. The treatments evaluated in this study differed in harvested intensity, spatial distribution of the skidding trails and width of residual strip as shown in Fig 2. Prescribed harvest intensity was 50 and 75% for shelterwood and ST, respectively [32]. MS consists of a succession of 5 m wide cut strips, with 5 m wide residual strips. In the case of CS and DS, trails are set at 20 m and 30 m intervals, respectively, and trees are partially harvested on each side of the trails, at a maximum distance of 5 m from the trail edge. DS presents secondary trails perpendicular to the main skidding trails and separated by 10 m. ST has wider 15 m cut strips with 5 m wide intact residual strips.

### Plot measurements

Rectangular sampling plots (10 × 60 m) covering the spatial heterogeneity of each treatment (trails, edge and residual strip) were established in the center of each experimental unit. The measurements were taken one year before cutting (b.c.) and 10 years after cutting (a.c.) on the trees with diameter at breast height (DBH) ≥9 cm. Two tree inventories were performed, the first one was general for all trees (n = 3739 and 2243, b.c. and a.c. respectively), and included species identification, DBH, wound severity (five levels from intact to major damage), tree state (dead or alive), and spatial position classes in the residual strip (edge and interior trees). The second inventory involved a subsample of randomly selected trees, and included tree height and crown length (n = 168 and 99, b.c. and a.c. respectively). The stand variables (density, mortality, basal area and dominant height) were estimated with the data of the inventories. Competition data were taken 10 years a.c. (n = 240). Hegyi’s index (CI_i_) was selected because it is the competition performance most strongly correlated with basal area growth in black spruce stands [28]. The distance (Dist_ij_) and DBH of each neighbour tree (_j_) within 4 m radius of the subject tree (_i_) were measured to calculate the CI_i_:

$$CI_{i}=\sum_{j=1}^{n}\left(\frac{DBH_{i}}{DBH_{J}}\times \frac{1}{Dist_{ij}}\right)$$ (1)

### Assessment of radial growth

In summer 2014, increment cores were extracted at breast height with a Pressler’s borer from 34 to 38 random trees per plot, resulting in 1039 sampled trees. The sampling was sized to efficiently represent the individual variation of growth in black spruce [43] and stratified on both edge and interior trees.

The samples were prepared, measured and analyzed following a standard dendroecological protocol [49]. Cores were air-dried, mounted on wooden boards and sanded. The tree rings were measured with WinDendro^™^ (version 2009, Regent Instruments, Quebec) or a manual Henson micrometer (LINTAB^™^, Rinntech, Heidelberg, Germany) with an accuracy of 0.01 mm [50]. The individual tree-ring series were cross-dated using TSAP-Win^™^ (Rinntech, Heidelberg, Germany) [51].

### Individual radial growth patterns

The nonlinear Schnute function was used to study the variability of individual radial growth [52] described by:

$$Y\left(t\right)={\left(\alpha +\beta e^{\gamma t}\right)}^{\delta }$$ (2)

where:

$$\alpha =y_{1}^{b}+\frac{\left(y_{2}^{b}/y_{1}^{b}\right)}{1-e^{-a\left(T_{2}-T_{1}\right)}}$$ (3)

$$\beta =\frac{e^{aT_{1}}\left(y_{2}^{b}-y_{1}^{b}\right)}{1-e^{-a\left(T_{2}-T_{1}\right)}}$$ (4)

γ=−a (5)

$$\delta =\frac{1}{b}$$ (6)

*T* is the time, *y*_*1*_ and *y*_*2*_ are the cumulative radial growth values at *T*_*1*_ and *T*_*2*_, corresponding to the cutting year and 10 years a.c., respectively, *a* is the constant in growth rate parameter, and *b* is the increment relative to *a*.

For each tree, individual models were fitted to the radial growth a.c., represented by the cumulative radial growth (CRG) since the treatment, calculated in terms of tree-ring width (mm). Model fitting was performed using nonlinear regressions. The analysis resulted in four curves representing different growth patterns according to the Schnute parameters:

Curve I (a>0, 0<b<1) is sigmoid, asymptotic and with an inflection point;
Curve II (a>0, b≥1) is asymptotic without inflection point;
Curve III (-b log(y_2_/y_1_)(t_2_–t_1_)<a≤0, 1< b) is S-shape, not asymptotic, but with an inflection point;
Curve IV (-b log(y_2_/y_1_)(t_3_–t_1_) <a≤0, 0≤b≤1) is exponential, not asymptotic, and without inflection point.

The accuracy of model fitting was evaluated using the root of weighted mean square error (RMSE).

### Factors influencing the growth patterns

We determined the differences in radial growth 10 years a.c. (*y*_*2*_) between growth patterns using an analysis of variance (ANOVA). Multiple and simple nominal logistic regressions were conducted to evaluate the relation between growth patterns and ecological or silvicultural traits based on structure type, stand age, harvested intensity, mortality, treatment, Hegyi’s competition index (CI_i_), dominant height, tree crown length, wound state, DBH and growth before cutting (GBC). A contingency analysis was performed to study changes in the frequency of growth pattern with structure, treatment and position. Step-wise regressions were used to identify the influencing factors on the radial growth response for each Schnute curve, represented by the cumulative tree-ring width 10 years a.c. When needed, a logarithmic transformation was performed to meet the assumption of homogeneity of variance and normality. The regression models were conducted for each study curve. Factors were selected by minimizing the minimum Bayesian Information Criterion (BIC). Multi-collinearity was verified on the predictors using the variance infraction factor (VIF) [53]. Eta^2^ was used for the estimation of the associated variance for each effect of the step-wise regressions [54]. All statistics were performed using JMP Pro statistical software, version 12 (SAS Institute Inc., Cary, NC).

## Results

### Individual radial growth patterns

**Fig 3:**
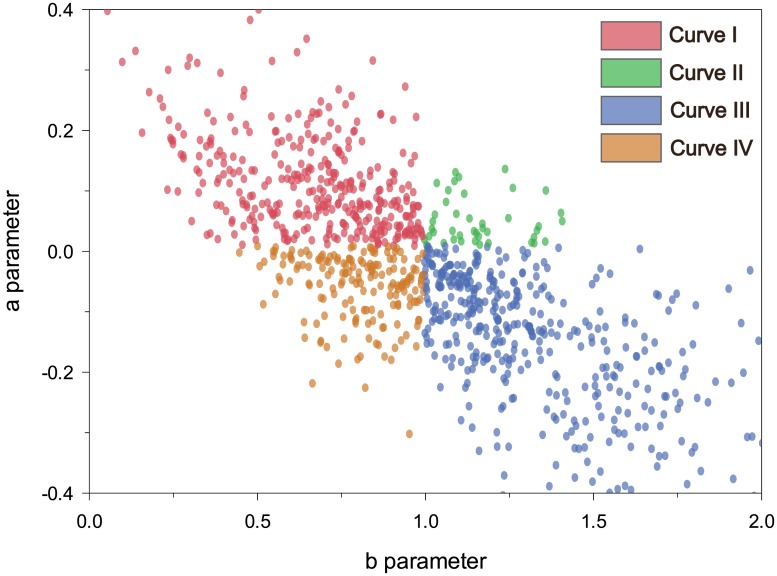
Distributions of the parameters of Schnute growth function for the trees sampled.

The Schnute function represented the growth of black spruce according to four patterns (Fig 3). Most trees (47%) were represented by Curve III (n = 489). Curves I and IV were observed in 32 and 17% of cases, respectively, while Curve II included only 4% of trees (n = 42). The Schnute parameters showed that the growth response a.c. was nonlinear (a≠0 and b≠1) in all the trees, and Curves I and III had the highest variability (Fig 3).

The accuracy of model fitting was adequate, as indicated by the high R^2^ (0.99 or higher), low RMSE (ranging from 0.05 to 0.12) and stable confidence limits (within ±0.02). The inflexion points identified for Curves I and III occurred between 5 and 9 years a.c., and around the first two years a.c., respectively. The horizontal asymptotes showed a high variation, and exhibited higher values for Curve I in respect to Curve II (Table 1 and S2 Appendix).

**Table 1:** Estimated parameters for Schnute curves. Values in parentheses represent the 95% confidence interval.

Parameter	Curve I		Curve II		Curve III		Curve IV	
a	0.10	(0.09–0.11)	0.04	(0.03–0.05)	-0.25	(-0.28 - -0.22)	-0.07	(-0.07 - -0.06)
b	0.66	(0.64–0.69)	1.16	(1.12–1.19)	1.68	(1.59–1.76)	0.80	(0.78–0.82)
y_2_	5.83	(5.33–6.34)	2.83	(2.16–3.50)	4.28	(4.03–4.53)	7.08	(6.48–7.69)
A	35.50	(26.02–45.00)	15.47	(9.69–21.25)	-		-	
y	12.82	(11.01–14.62)	-		3.58	(2.53–4.64)	-	
t	7.46	(5.19–9.73)	-		1.02	(0.75–1.30)	-	
RMSE	0.10	(0.09–0.12)	0.05	(0.03–0.07)	0.07	(0.06–0.07)	0.12	(0.10–0.14)
R^2^	0.99	(0.996–0.997)	0.99	(0.995–0.997)	0.99	(0.996–0.997)	0.99	(0.997–0.998)
N	336		42		489		172	

a, b, y_2_ –Schnute parameters, A—asymptote, y, t—coordinates of the inflexion point, RMSE—root mean square error of estimation, N- number of trees.

The Schnute curves showed important and significant differences in growth 10 years a.c. (p<0.0001). Curve IV had the highest radial growth, with 7.08 mm (*y2* parameter, Table 1). The least growth (2.83 mm) was observed for Curve II, three times lower than for Curve IV. Curves I and III showed intermediate growth of 5.83 and 4.28 mm, respectively.

**Fig 4:**
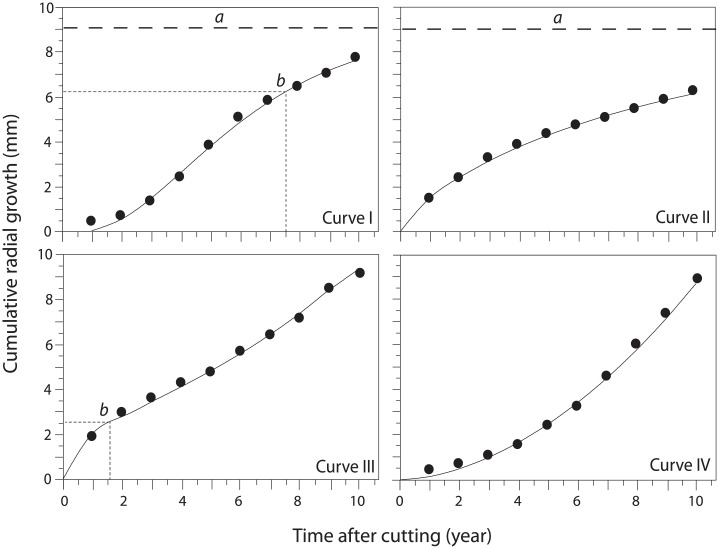
Examples of the four growth patterns using the Schnute function. The discontinuous horizontal lines indicate the asymptotes (a). The thin discontinuous horizontal and vertical lines show the inflexion points (b). The black dots show the cumulative radial growth values for each study year and the continuous lines represent the model fitting.

The growth dynamics differed between studied curves (Fig 4). Curve I showed a slow growth lasting up to 3 years, a growth increase of 4–7 years followed by a growth decrease. Curve II was characterized by a reduced and almost constant growth of 0.2–0.3 mm year^-1^. Curve III displayed a phase of fast growth increase lasting up to 3 years, followed by a small decrease until 7 years a.c., after which growth again started to increase. Curve IV had an exponential growth of 0.6–0.8 mm year^-1^.

### Factors influencing the growth patterns

**Fig 5:**
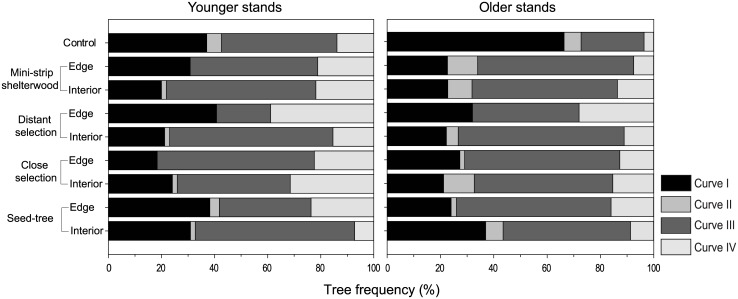
Frequency of growth patterns represented by structure, treatment and spatial position in the residual strip.

Multiple nominal logistic regression determined that the frequency of each growth pattern varied between younger and older stands (p = 0.0009), and among silvicultural treatments and position classes (p<0.0001). Curve I represented the growth pattern of 66 and 40% of control trees in older and younger stands, respectively (Fig 5). Little differences were observed among silvicultural treatments, Curve I varying from 23% in CS to 29% in ST. The effect of position was detected in younger stands, with Curve I being 10–20% more frequent in edge than interior trees. Curve II was three times more common in older than younger stands (6 and 2% respectively), and was sporadic in edge trees and prominent in interior trees. Curve III was less present in the control (34%) than treated trees (45% in DS to 62% in CS). Curve IV was more frequent in younger stands (21%) and edge trees (22%) than in older stands (12%) and interior trees (13%). The lowest frequency of Curve IV was found in control trees of older stands (4%) and the highest in the DS edge trees in younger stands (39%).

**Fig 6:**
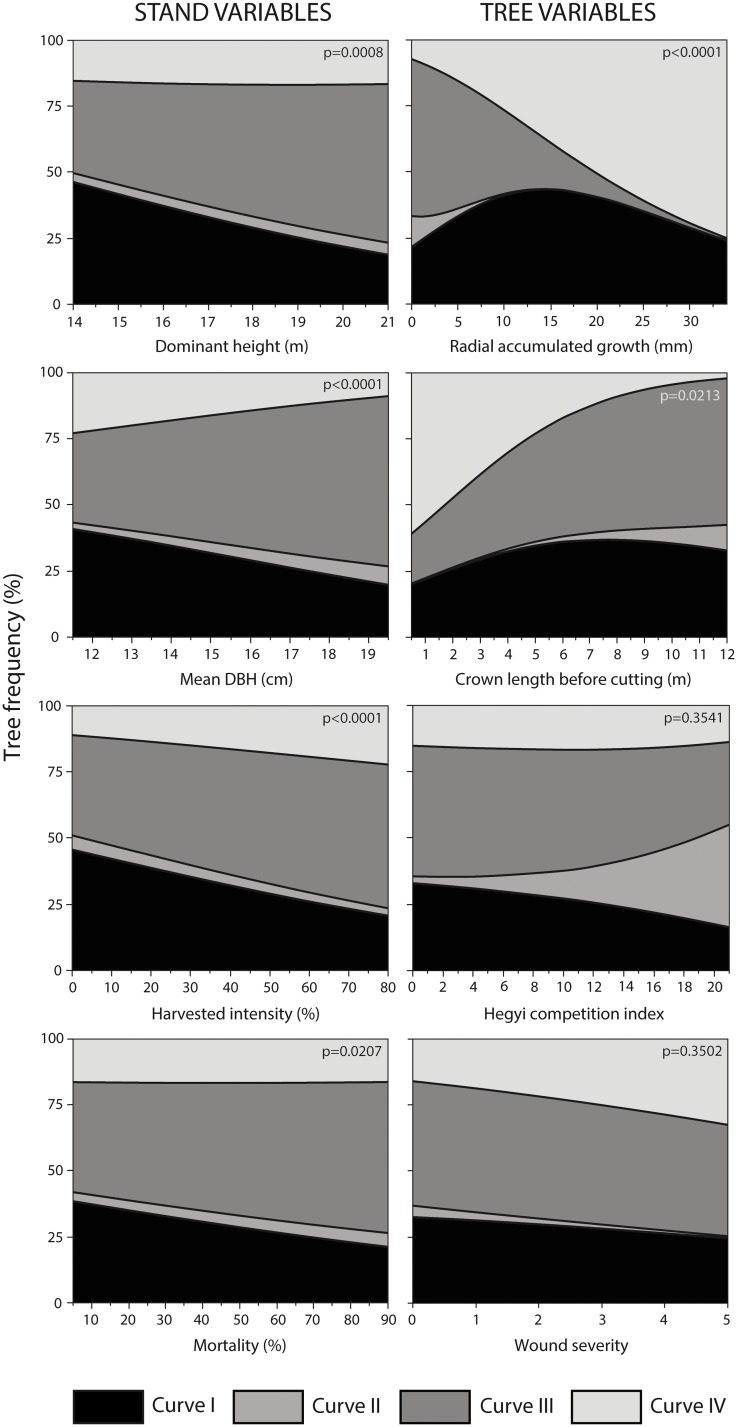
Variation in the frequency of Schnute curves according to different stand and tree variables.

The simple nominal logistic regressions described how the concurrency of the curves changed according to the stand and tree variables (Fig 6). Dominant height, DBH, mortality and harvested intensity were the most significant stand variables (p<0.05). The growth response for 50% of trees was explained by Curve I in stands with low dominant height, DBH, mortality and harvested intensity, while Curve III represented the growth response for 60% of trees in stands with high values of these variables. The occurrence of Curve IV doubled in stands with low DBH and high harvested intensity. Generally, Curve II registered low variation in tree frequency, and was more common in stands with high DBH and low harvested intensity (Fig 6).

CRG was the tree variable that most influenced the frequency of curves (p<0.0001). Sixty percent of trees with over 25 mm of CRG 10 years a.c. were represented by Curve IV. On the contrary, the smallest radial growth response was detected by Curve II, because no tree with CRG value above 10 mm was found for this curve. Curves I and III showed intermediates CRG values, 31 and 9% of trees respectively exhibited a radial growth ≥25 mm. Crown length b.c. was a significant variable to explain the Schnute curves in the studied trees (p<0.05). Curve II was sporadic at low values of crown length (<5m). Circa 60% of trees with low values of crown length b.c. were associated with Curve IV, while ≤5% of trees with high values were represented by this curve. Curve III was three times more frequent in trees with high values of crown length (Fig 6). Curve I was more common in trees with intermediate crown length b.c.

The majority of tree variables a.c. were not significant (p>0.05) contrary to b.c. e.g. DBH, height, crown length, wound severity and CI_i_. However, these variables vary with the tree curve frequencies and showed different trends, especially CI_i_ and wound severity (Fig 6). The CI_i_ determined that the growth response of 30% of trees with low competition was represented by Curve I, and only 15% with high values of competition. Curve II represented well the growth response of trees with high CI_i_ (40%), but poorly the growth response of trees with low CI_i_ (<5%). Wound state, identified that Curve IV was more frequent at higher damage, being the growth response for 40% of trees with severe wounds and 15% of intact trees (three times lower). Curve II was rarely represented in wounded trees, being almost inexistent for severely injured trees.

**Fig 7:**
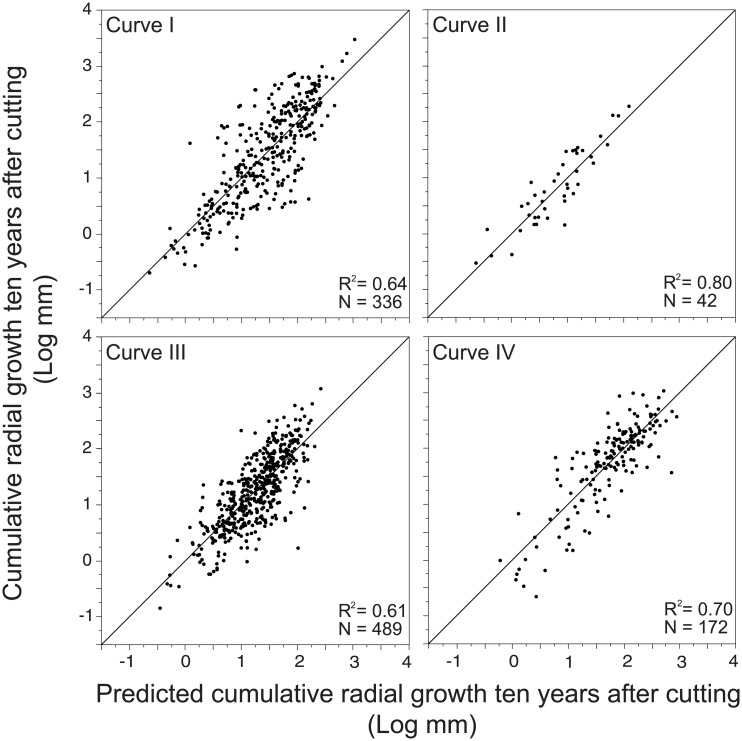
Observed vs. predicted growth ten years after cutting modelled by growth pattern.

The variance explained of CRG 10 years a.c. by the multiple linear regressions (R^2^) ranged from 61 to 80%; residual plot distributions indicated adequate fitting (Table 2; Fig 7). These analyses evidenced that GBC was the most important factor explaining the variations in growth response 10 years a.c. for all the studied curves (p<0.0001), although the spatial position, stand structure, age and mortality were secondary variables. Curve II had the highest R^2^, and was the simplest model with the variation explained by only one factor (GBC). The most complex growth pattern was observed for Curve III, with six factors involved in the ecological explanation, and the lowest fitting detected in the studied curves; This growth pattern was identified in interior trees of residual stands with high harvested intensity, older age-structure, high dominant height and high mortality rates (Table 2). Curves I and IV showed intermediate fitting, with R^2^ of 0.64 and 0.70, respectively and four retained factors. In the case of Curve I, treatment and age were the most important determinant factors explaining the growth response. The growth of trees that followed the pattern of Curve IV was higher in edge trees of younger stands and positively related to dominant height and GBC (Table 2).

**Table 2:** Stepwise regressions for the cumulative radial growth of black spruce for each curve of the Schnute function using the forward procedure with Bayesian Information Criterion (BIC) as indicator.

Curve	R^2^	N	Parameters	Estimate±Sd error	t	VIF	p-value	Eta^2^
I	0.64	336	treatment	0.10**±**0.03	-3.00	1.34	0.0029	0.0131
			position	0.08**±**0.03	2.54	1.24	0.0114	0.0052
			stand age	0.19**±**0.03	-5.80	1.29	<0.0001	0.0662
			GBC	0.84**±**0.05	16.82	1.21	<0.0001	0.3023
II	0.80	42	GBC	0.99**±**0.07	13.00	1	<0.0001	-
III	0.61	489	treatment	0.13**±**0.03	-3.97	1.26	<0.0001	0.0132
			position	0.04**±**0.02	2.12	1.18	0.0342	0.0039
			structure	0.08**±**0.02	3.98	1.20	<0.0001	0.0129
			dominant height	0.04**±**0.01	2.66	1.06	0.0081	0.0056
			mortality	0.05**±**0.02	2.56	1.23	0.0108	0.0029
			GBC	0.79**±**0.03	23.53	1.13	<0.0001	0.4330
IV	0.70	172	position	0.07**±**0.03	2.38	1.00	0.0185	0.0101
			structure	0.11**±**0.04	3.10	1.23	0.0023	0.0172
			dominant height	0.06**±**0.02	2.75	1.12	0.0066	0.0135
			GBC	0.86**±**0.05	17.40	1.12	<0.0001	0.5421

VIF = Variance Inflection Factor, GBC = growth before cutting.

## Discussion

### Determination of growth patterns with individual nonlinear models

This study proposes an application of the Schnute function [52] to describe the inter-individual variations of tree growth response after partial cutting in black spruce stands. Due to their flexibility, the equations have confirmed their ability to emulate a large spectrum of forest growth dynamics [18–21, 55], and their easy fit and quick convergence [18]. In forest management, this model was used to study height-diameter relationship [15, 18, 19, 56], individual tree basal area growth [20] and the sequestration of atmospheric carbon [57]. However, to our knowledge this is the first time that the Schnute function have been used with tree-ring series characterizing individual growth responses to partial cutting.

The Schnute nonlinear model described the heterogeneity of tree growth according to four patterns. Each growth curve differed in form, and complexity, and represented the variability in the response of trees to the treatments and the ecological factors involved in growth. Curves I and III were the most representative for their sigmoid form, the well-known shape of growth response, although the inflection points showed a certain variability. Curve IV was the simplest pattern due to its exponential shape and the absence of inflection points or asymptotes, and showed the highest values in cumulative radial growth after treatment. Curve II showed the lowest growth responses, represented by a low horizontal asymptote. However, the asymptote is the least stable parameter, especially in the case of few nearby sampling points [17] and this curve showed the highest dispersion and variation in the mean cumulative growth, probably due to the low numbers of trees. Certain traits should be considered to select the growth functions: monotonic increment, inflection point, horizontal asymptote and flexibility [56]. All these elements are easily estimated by the Schnute models, which have been demonstrated to be a useful and versatile function for forestry applications.

Previous studies conducted in the eastern Canadian boreal forest showed that black spruce growth after partial cutting follows three steps: (i) no response during two to five years after treatment; (ii) a rise in growth during 10 years after cutting; and (iii) a progressive return of the growth increment to that observed before treatment [32, 42, 43, 58]. Our study shows that this pattern, which corresponds to Curve I, is observed in only 32% of residual trees, and could explain why a high proportion of the growth variability remained unexplained in previous investigations based on linear stand approaches [25, 26, 32, 34, 43, 58]. We demonstrated that the response of black spruce is heterogeneous among trees, and, in addition to the sigmoid functions (represented by Curves I and III), a part of the variability was due to the presence of other growth patterns (represented by Curves II and IV).

### Factors influencing the occurrence of growth patterns

The ecological interpretation is an important element to consider [24], because biologically reasonable models generally produce more accurate predictions [8, 59]. Until now, all studies on black spruce have attributed the large unexplained variation (around 50%) in growth to variability among trees [25, 34, 43] assuming the same growth pattern for all trees within a stand. With this approach, the driving factors of individual variability were never taken into account. Our new approach shows the diversity in growth response among trees, and was able to explain 61–80% of variation in cumulative radial growth 10 years a.c. for studied curves, identifying the factors potentially involved in the occurrence of each pattern. GBC was the most important variable to explain the variation in growth response, as in other recent investigations [25, 32], although spatial position, stand structure, age and mortality play an essential role in the studied curves. We raise the hypothesis that the remaining unknown variability (20–39%) could be caused by microclimate or tree age, and we recommend considering these factors more carefully in future research. Similarly, we propose an applied individual nonlinear modeling to explain the heterogeneity and diversity of growth patterns after partial cutting.

Curve III represented most residual trees (ca 50%), mainly the interior ones in DS and MS, and was related to stand density, structure and mortality, as shown in other studies [60–62]; Within the residual band, tree growth is affected by the competition for resources [24]. We explained this pattern with the temporal growth response detected. This sigmoid curve is characterized by an inflexion point at 1–2 years a.c. similar to the results obtained by Bredenkamp and Gregoire [60]; the interior trees experienced low growth increase until two years a.c. The stabilization in the growth rate (3–7 years a.c.) could be because the removal of neighbour trees in the residual strip was not strong, being harvested only 33% of trees in DS and CS. We thus hypothesize that with high harvested intensity (e.g. around 50% of interior trees) the growth response could be higher and last longer. The enhanced in growth registered at 7–10 years a.c. was explained by the competition and mortality relationship in previous studies [61, 62]. In our case, it could be related to the reduction in competition due to the mortality of residual trees by windthrow, which usually occurs between 0 and 5 years after treatment[63, 64].

Curve I, the second predominant growth pattern, was associated with the control trees of older stands. However, the difference between stand structures may be explained by a windthrow event in the year of cutting that affected two younger control plots. Consequently, we argue that the true difference according to the experimental treatments has been exaggerated by this disturbance. Windthrow caused a reduction in stand density, and this could influence the growth response. These events are common in our study area, 20–30% of trees were affected by windthrow in the period 2006–2010, so our results are close to the real influence of natural disturbances [65]. The position effect influenced this growth pattern in younger stands, being more frequent in edge trees, especially in DS and MS. We hypothesize that some edge trees experienced a stress period during the first years after cutting due to root damage or instability by wind exposure [66, 67], which resulted in a slower growth rate between 0 and 5 years a.c. In older stands, the edge effect is less than in younger stands [32, 68], this could explain why the differences between edge and interior trees was less obvious. Moreover, the growth response to partial cutting decreases with the age, with fewer released trees in older stands [26].

Curve IV showed an exponential increase exhibited principally in edge trees (40% in DS of younger stands) and trees that were suppressed before the treatment. This curve confirmed why the radial growth response of edge trees is greater than interior trees [32, 69]. Curve IV was more frequent in younger stands. Accordingly, the release of suppressed trees is more important in high density stands and edge effect was stronger in younger stands with high harvested intensity [32]. Partial cuttings favour suppressed trees due to reduced competition for light, water and nutrients [34], this could explain the high growth response detected. This curve showed the best performance in growth, but was detected in only 25% of trees in younger stands. The Schnute Curve IV could be used as an indicator for effectiveness of the treatments in terms of radial growth. Also, promoting the edge surface in younger stands during the silvicultural planning can maximize this pattern after partial cuttings in the context of boreal forest management.

Curve II, with a constant growth decline was the least represented (4% of trees) in residual trees, and was characterized by the lowest cumulative growth values (50% less than Curve I). This curve represented the growth response of trees that were not released after cutting. Partial cuttings significantly reduced the occurrence of this pattern in younger stands, although this was not as clear in older stands. This can be explained by the growth decrease with age, it being more likely to find old trees with low growth responses [42]. The low CRG values detected in this curve could also be because this pattern was most usual in stands with high CI_i_ and low height, thus it may represent suppressed trees that were insensitive to the treatments.

In even-aged black spruce stands, the long-term diameter growth trend generally follows a classical sigmoid pattern [70]. In this case, the Schnute growth curves covering a window of 10 years can be associated to the different growth phases during the tree life cycle: Curve IV may be linked to the juvenile exponential growth phase, followed in time by Curves III and I, where the growth slows down and creates an inflexion point in the growth curve, while Curve II represents the last growth phase where diameter growth shows a progressive reduction over time (Fig 4) [32]. Thus, growth patterns change among life stages of trees and with ecological conditions (tree status, competition, spatial position in the stand…). This interpretation is supported by the higher representation of Curves III and IV in young control plots, and the higher proportion of Curves I and II in older controls. The higher proportion of Curve I in younger stands than older ones may be caused by the presence of suppressed trees that died by self-thinning over time [71], and from a windthrow event in one of the younger control plots. Our study evaluated the growth response 10 years a.c., although a longer monitoring could better estimate the growth patterns of trees.

We demonstrated that dominant height, DBH, mortality, harvested intensity and age structure affect growth after treatment in different silvicultural scenarios. These variables could therefore be good indicators for stand selection to maximize radial growth after partial cutting. Some of the variables identified in the occurrence of growth patterns were studied and considered valid to predict the growth responses in previous nonlinear model studies, e.g. dominant height was used in a lot of height-diameter models [8, 24, 72]; the influence of stand age in the growth response a.c. was studied by Thorpe in 2007; stand density was considered the most obvious factor influencing the height-diameter relationship [8, 16, 18, 73]; tree initial DBH was one of explanatory variables used to predict the basal area periodic increment in black spruce stands by Zhang et al. 2004; and harvested intensity was identified as a determinant of the magnitude and duration of the growth response [73]. In the case of CI_i_, trends could be perceived but could not be statistically confirmed, likely due to the small sample size.

## Conclusions

Our innovative approach evidenced the different growth patterns of trees that could reduce the variance unexplained by the classical models. The Schnute function has been demonstrated to have a great potential for describing the individual response of trees at fine temporal scales, and obtaining a high resolution of growth analysis with dendrological data. Within the stand, heterogeneous growth has to be accurately assessed and ecologically interpreted to correctly evaluate the effects of partial cuttings on the trees. Consequently, all knowledge that allows an understanding of how the trees can respond to a treatment is useful, and the Schnute function could be an analytic support in ecosystem management of the boreal forest.

## Supporting information

S1 Appendix Schematic representation of the experimental design. Stand structures (younger and older) are indicated as supra-variable in the design. The blocks are numbered (1 to 6), the experimental units are capital letters: study treatments (A-B-C-D) and control plots (E) and, the position classes of trees are lowercase letters: edge (e) or interior (i) and the quantity of tree samples by position class are indicated by the corresponding number.
(PDF)

S2 Appendix Inter-annual variability in cumulative radial growth for the four Schnute curves. The continuous lines show the mean values and the discontinuous lines indicate the lower and upper 95% confidence intervals.
(PDF)
