# Supplementary material for: Understanding tree growth responses after partial cuttings: A new approach
Source: PLoS One. 2017 Feb 21;12(2):e0172653. doi: 10.1371/journal.pone.0172653 (PMC5319695; doi:10.1371/journal.pone.0172653)

S1 Appendix. Schematic representation of the experimental design. Stand structures (younger and older) are indicated as supra-variable in the design. The blocks are numbered (1 to 6), the experimental units are capital letters: study treatments (A-B-C-D) and control plots (E) and, the position classes of trees are lowercase letters: edge (e) or interior (i) and the quantity of tree samples by position class are indicated by the corresponding number.

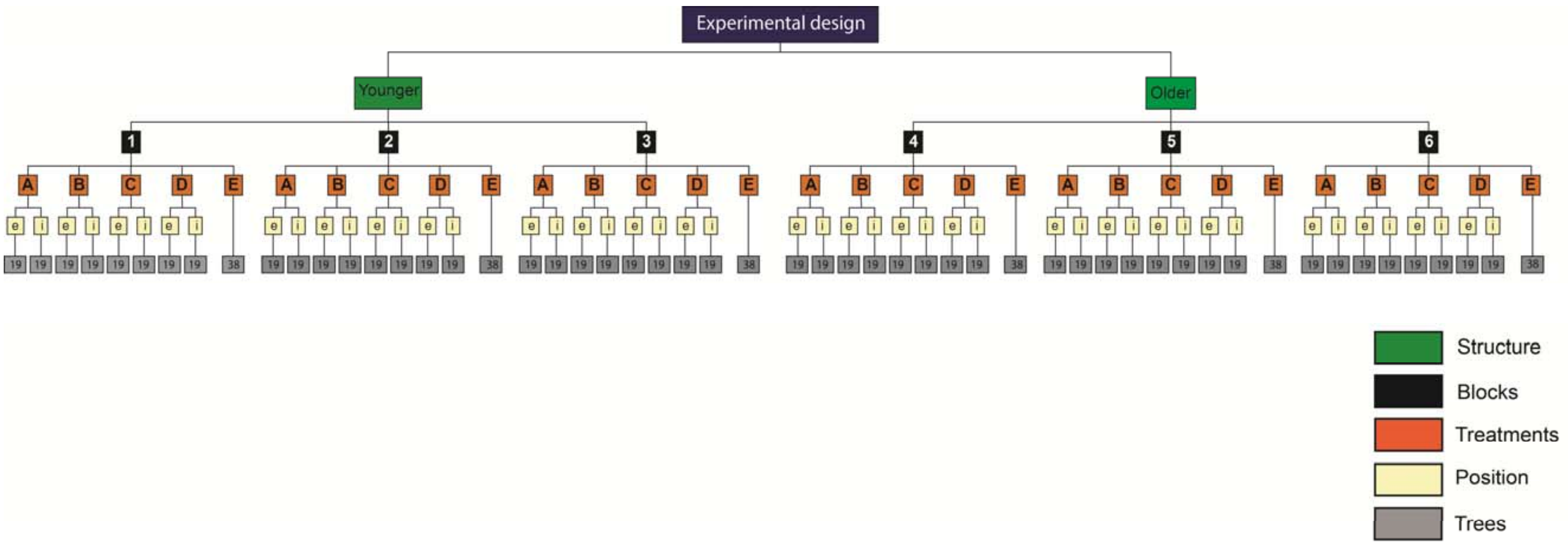

Supplement: S1 Appendix — Stand structures (younger and older) are indicated as supra-variable in the design. The blocks are numbered (1 to 6), the experimental units are capital letters: study treatments (A-B-C-D) and control plots (E) and, the position classes of trees are lowercase letters: edge (e) or interior (i) and the quantity of tree samples by position class are indicated by the corresponding number. (PDF) [file pone.0172653.s001.pdf]
