# Supplementary material for: Understanding tree growth responses after partial cuttings: A new approach
Source: PLoS One. 2017 Feb 21;12(2):e0172653. doi: 10.1371/journal.pone.0172653 (PMC5319695; doi:10.1371/journal.pone.0172653)

S2 Appendix. Inter-annual variability in cumulative radial growth for the four Schnute curves. The continuous lines show the mean values and the discontinuous lines indicate the lower and upper 95% confidence intervals.

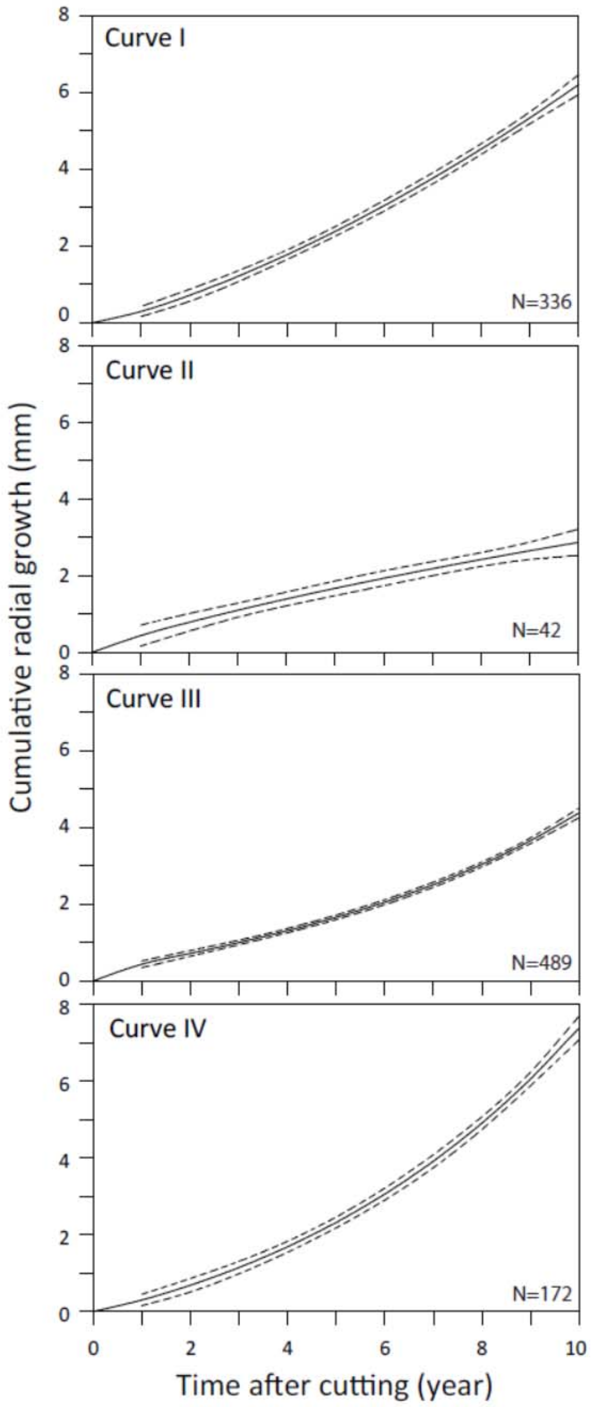

Supplement: S2 Appendix — The continuous lines show the mean values and the discontinuous lines indicate the lower and upper 95% confidence intervals. (PDF) [file pone.0172653.s002.pdf]
